# Colon surgical-site infections and the impact of “present at the time of surgery (PATOS)” in a large network of community hospitals

**DOI:** 10.1017/ice.2022.236

**Published:** 2022-09-22

**Authors:** Jessica L. Seidelman, Maojun Ge, Arthur W. Baker, Sarah Lewis, Sonali D. Advani, Becky Smith, Deverick J. Anderson

**Affiliations:** 1 Division of Infectious Diseases and International Health, Department of Medicine, Duke University School of Medicine, Duke University, Durham, North Carolina, United States; 2 Duke Center for Antimicrobial Stewardship and Infection Prevention, Duke University Medical Center, Durham, North Carolina, United States; 3 Department of General Surgery, Shuguang Hospital, Shanghai University of T.C.M., Pudong, Shanghai, China

## Abstract

**Objectives:**

To describe the epidemiology of complex colon surgical procedures (COLO), stratified by present at time of surgery (PATOS) surgical-site infections (SSIs) and non-PATOS SSIs and their impact on the epidemiology of colon-surgery SSIs.

**Design:**

Retrospective cohort study.

**Methods:**

SSI data were prospectively collected from patients undergoing colon surgical procedures (COLOs) as defined by the National Healthcare Safety Network (NHSN) at 34 community hospitals in the southeastern United States from January 2015 to June 2019. Logistic regression models identified specific characteristics of complex COLO SSIs, complex non-PATOS COLO SSIs, and complex PATOS COLO SSIs.

**Results:**

Over the 4.5-year study period, we identified 720 complex COLO SSIs following 28,188 COLO surgeries (prevalence rate, 2.55 per 100 procedures). Overall, 544 complex COLO SSIs (76%) were complex non-PATOS COLO SSIs (prevalence rate [PR], 1.93 per 100 procedures) and 176 (24%) complex PATOS COLO SSIs (PR, 0.62 per 100 procedures). Age >75 years and operation duration in the >75th percentile were independently associated with non-PATOS SSIs but not PATOS SSIs. Conversely, emergency surgery and hospital volume for COLO procedures were independently associated with PATOS SSIs but not non-PATOS SSIs. The proportion of polymicrobial SSIs was significantly higher for non-PATOS SSIs compared with PATOS SSIs.

**Conclusions:**

Complex PATOS COLO SSIs have distinct features from complex non-PATOS COLO SSIs. Removal of PATOS COLO SSIs from public reporting allows more accurate comparisons among hospitals that perform different case mixes of colon surgeries.

Surgical site infections (SSIs) are the most common healthcare-associated infection (HAI) in the United States and account for almost a quarter of all HAIs.^1,2^ Approximately 20% of SSIs in the United States occur following colon surgery.^3-6^

Surveillance and feedback of SSI rates is a critical component of SSI prevention activities.^7^ In 2015, the Centers for Disease Control and Prevention’s (CDC) National Healthcare Safety Network (NHSN) introduced a required element for SSI surveillance, “present at time of surgery” (PATOS).^8^ When SSIs occur following index surgical procedures in which infection at the same depth was visualized and documented in the operative note, these SSIs are deemed PATOS. Beginning in 2017, PATOS SSIs were excluded from the Centers for Medicare and Medicaid Services (CMS) colon (COLO) SSI standardized infection ratio (SIR), a measure used as part of public reporting and federal hospital reimbursement programs.^9^

Since the introduction of this definition, data characterizing PATOS colon SSIs^10^ and the impact of excluding PATOS colon SSIs from CMS reporting have been limited. The objectives of this study were to describe the epidemiology of colon SSIs, to compare characteristics between PATOS colon SSIs and non-PATOS colon SSIs, and to describe the impact of excluding PATOS colon surgery SSIs from CMS reporting.

## Methods

### Setting

We performed a retrospective cohort analysis of COLO SSI surveillance data from 34 community hospitals in the southeastern United States participating in the Duke Infection Control Outreach Network (DICON) from January 2015 to June 2019. The DICON network offers infection control consultation, data analysis and support, and education to community hospitals.^11^ Board-certified infection preventionists (IPs) prospectively gather data from the DICON Surgical Surveillance database, which contains data from acute-care hospitals performing 37 types of operative procedures. The database contains patient- and surgery-specific variables: type of surgical procedure, hospital, primary surgeon, patient age, procedure date and duration, NHSN risk index (calculated from the patient’s American Society of Anesthesiologists [ASA] classification system score, wound class, and operative duration), and the presence or absence of postoperative SSI for all procedures. When an SSI occurs, the type of SSI, date of diagnosis, and causative organism (if a postoperative culture was obtained and was positive), are added. DICON surveillance methods have been described previously.^12^

Since 2015, the PATOS designation has been applied in the database according to the NHSN definition, “evidence of infection visualized (seen) during the surgical procedure to which the subsequent SSI is attributed.”^9^ To meet criteria, IPs determine whether evidence of infection was noted intraoperatively at the same depth as the subsequent SSI and documented within the narrative portion of the operative note.

### Study population

We identified surgical procedures for the study using the DICON surgical surveillance database. Hospitals were included in the analysis if complete COLO surveillance was available for the study period. All procedures with a “COLO” label, as defined by NHSN,^9^ were eligible for inclusion. We limited the analysis to complex COLO SSIs, defined as deep incisional or organ-space SSIs.^9^ We excluded surgeries performed on patients aged <18 years at the time of surgery.

### Objective

The primary objective of our study was to describe the epidemiology of complex COLO SSIs, stratified by PATOS and non-PATOS SSIs. Specifically, we wanted to examine the complex COLO SSI rate, presented as prevalence rate (number of SSIs per 100 procedures) and stratified by PATOS colon SSIs and non-PATOS colon SSIs.

### Analysis plan

First, we analyzed the prevalence rate of complex COLO SSIs following colon surgeries from January 2015 to June 2019. Specifically, we evaluated trends in the rate of complex COLO SSIs over the study period, stratifying the cases by PATOS versus non-PATOS category. We also described the prevalence of pathogens that caused COLO SSIs.

Second, we examined variables associated with COLO SSIs. We compared these characteristics between surgeries with complex COLO SSIs and surgeries without COLO SSIs using variables previously identified in the literature, including age, sex, body mass index (BMI), operation duration in minutes, prior diabetes diagnosis, American Society of Anesthesiologists (ASA) physical status, wound class, emergent procedure, endoscopic procedure, and hospital volume of colon surgeries performed. Based on the median hospital volume of 500 COLO procedures performed, we defined a low hospital volume as <500 COLO procedures performed during the 4.5-year study period. Variables were summarized using descriptive statistics. First, we compared variables between the surgeries with and without complex COLO SSIs using *t* tests and χ^2^ tests. A 2-sided *P* value < .05 was considered statistically significant.

Third, we described and compared the same a priori variables between COLO surgeries with PATOS COLO SSIs and non-PATOS COLO SSIs. We compared variables between these 2 groups using the same descriptive statistics outlined above.

Fourth, we constructed multivariable logistic regression models to compare characteristics from prior methods of reporting, which included PATOS COLO SSIs, to characteristics using current methods of reporting, which only included non-PATOS COLO SSIs. For all models, we included the previously mentioned a priori variables. However, we converted the following continuous variables to binary variables to preserve power: age >75 years, body mass index (BMI) >30 kg/m^2^, and surgery duration >75th percentile in the cohort (187 minutes).

Fifth, we constructed 3 multivariable models with different outcomes of interest. The outcome of interest in model 1 was all complex COLO SSIs; the outcome of interest in model 2 was non-PATOS complex COLO SSIs; and the outcome of interest in model 3 was PATOS complex COLO SSIs. We considered variables to be significant if the odds ratios from the multivariable logistic regression model had a *P* value < .05.

Lastly, we used a likelihood ratio test to evaluate for effect measure modification (EMM) between the variables emergent surgery and hospital volume, laparoscopic surgery and hospital volume, contaminated or dirty wound class and hospital volume, and emergent surgery and contaminated or dirty wound.

The Duke University Health System Institutional Review Board approved this research project. We analyzed all data using SAS version 9.4 software (SAS Institute, Cary, NC).

## Results

**Fig. 1.**
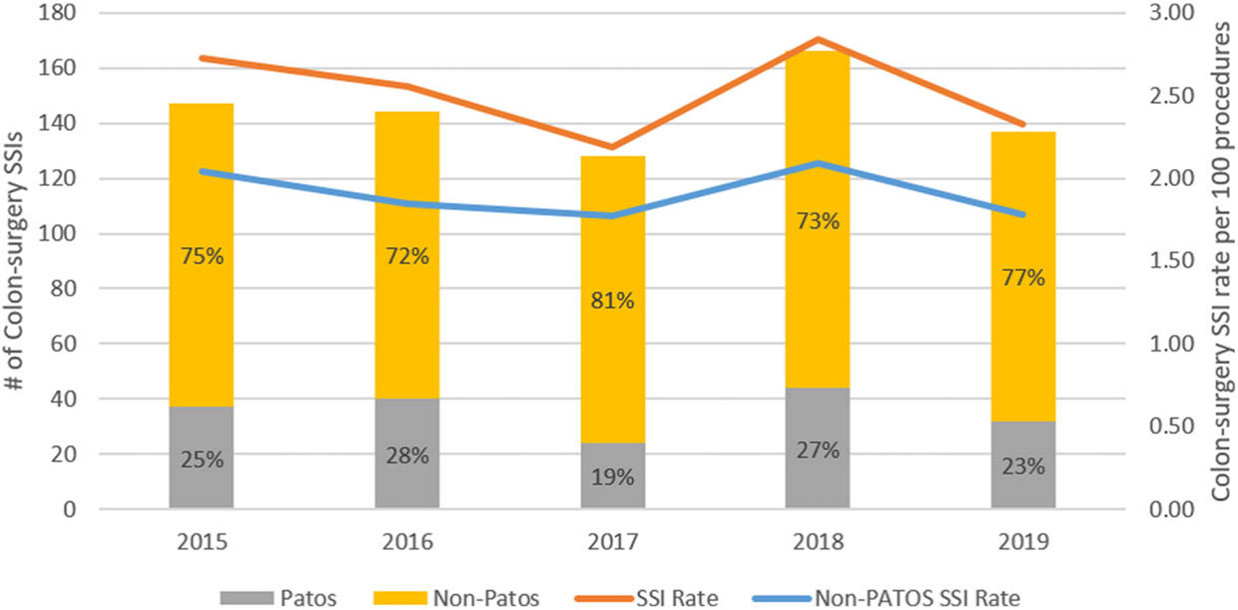
Prevalence of complex colon surgery SSIs from 2013 to 2018, stratified by PATOS status.

We identified 720 complex COLO SSIs following 28,188 COLO surgeries during the 4.5-year study period (prevalence rate [PR], 2.55 per 100 procedures). The rate of complex SSI did not change meaningfully during the study period (Fig. 1).

The average age of patients with complex SSI was 54.2 years (SD, 14.9), and 356 (49%) were male. Compared to patients without complex COLO SSI, patients with complex COLO SSI were more likely to have prolonged surgical duration, contaminated or dirty wound class, an emergency procedure, and an open surgery (Table 1).

Overall, 544 complex COLO SSIs (76%) were complex non-PATOS COLO SSIs (PR 1.93 per 100 procedures) and 176 (24%) were complex PATOS COLO SSIs (0.62 per 100 procedures). The proportion of PATOS COLO SSIs ranged from 19% to 28% over the study period (Fig. 1). In univariate analyses comparing patients with non-PATOS COLO SSIs to patients with PATOS COLO SSI, patients with PATOS COLO SSIs were more likely to have contaminated or dirty wound class, an emergency procedure, an open surgery, and surgery performed in a hospital with a high surgical volume (Table 2).

We then analyzed 3 multivariable logistic regression models to identify independently associated factors for complex COLO SSIs, complex non-PATOS COLO SSIs, and complex PATOS COLO SSIs, which we refer to as models 1, 2, and 3, respectively. In the multivariable analysis of model 1, we compared patients with complex COLO SSIs to patients without complex COLO SSIs. Independently associated factors for complex COLO SSI in our cohort included age >75 years (odds ratio [OR], 1.56; 95% confidence interval [CI], 1.23–1.96), operation duration >187 minutes (OR, 1.75; 95% CI, 1.50–2.04), contaminated or dirty wound class (OR, 1.27; 95% CI, 1.07–1.50), emergency procedure (OR, 1.87; 95% CI, 1.53–2.29), open surgery (OR, 1.20; 95% CI, 1.03–1.39), and hospital volume >500 procedures during the study period (OR, 1.35; 95% CI, 1.08–1.69) (Table 3, model 1).

In model 2, we compared patients with complex non-PATOS COLO SSIs to patients without complex non-PATOS COLO SSIs. Variables independently associated with complex non-PATOS COLO SSIs included age> 75 years (OR, 1.37; 95% CI, 1.06–1.75), operation duration >187 minutes (OR, 1.82; 95% CI, 1.53–2.16), and contaminated or dirty wound class (OR, 1.28; 95% CI, 1.03–1.60) (Table 3, model 2).

In model 3, we compared patients with and without complex PATOS COLO SSIs. Significant variables from this model included contaminated or dirty wound class (OR, 2.80; 95% CI, 2.12–3.71), emergency surgery (OR, 1.88; 95% CI, 1.45–2.44), and hospital volume >500 surgeries (OR, 2.02; 95% CI, 1.22–3.33) (Table 3, model 3).

**Fig. 2.**
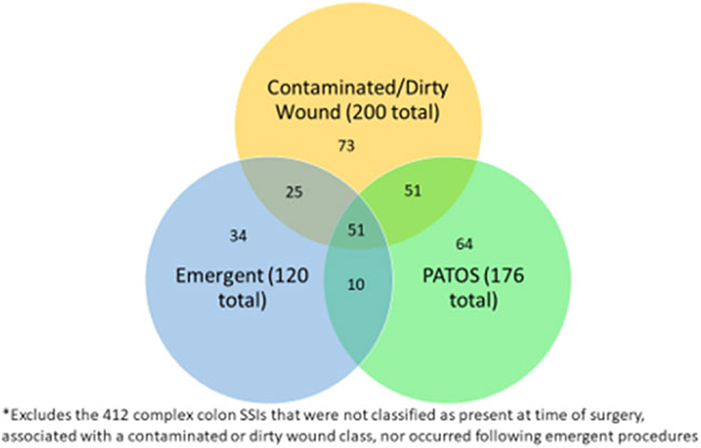
Categorization of 308 complex colon SSis by contaminated or dirty wound class, present at time of admission (PATOS), and emergent procedure.

Among the 200 complex COLO SSIs that were classified as having a contaminated or dirty wound class, 76 (38%) occurred after emergent procedures and 102 (51%) were identified as PATOS. Of these 76 complex COLO SSis that were emergent procedures and had a contaminated or dirty wound class, 51 (67%) were described as PATOS (Fig. 2). Conversely, of the 176 complex PATOS COLO SSis, 61 (35%) were emergent procedures, 102 (58%) had contaminated or dirty wound class, and 51 (29%) were emergent and had contaminated or dirty wound class. Likelihood ratio tests found no significant effect measure modification between the emergent surgery and hospital volume variables (*P* = .31), laparoscopic surgery and hospital volume variables (*P* = .41), or contaminated or dirty wound and hospital volume variables (*P* = .13). However, a significant interaction was present between contaminated or dirty wound class and emergent surgery (*P* < .01).

Of the 2,774 emergent procedures in the data set, 2,042 (73.6%) were performed at high-volume centers. Furthermore, 2,076 (74.5%) of the emergent procedures were open procedures. Lastly, 4,838 (81.5%) of the contaminated or dirty wounds were seen at high-volume centers.

The distribution of pathogens among complex COLO SSis consisted of typical enteric flora (Table 4). The proportion of specific organisms among PATOS COLO SSis and non-PATOS colon SSis was similar. However, the proportion of polymicrobial SSis was higher among complex non-PATOS COLO SSis compared to complex PATOS COLO SSis (0.69 vs 0.33; *P* < .01).

## Discussion

This 4.5-year, multicenter, retrospective cohort study is the largest to describe the impact of PATOS SSI on the epidemiology of colon SSis. Removing PATOS eliminated ~25% of complex colon SSis that would have previously been attributed to hospitals. Different variables were associated with PATOS and non-PATOS SSis, suggesting that patients and procedures that lead to COLO SSis characterized as PATOS have different characteristics than patients and procedures that lead to COLO SSis not characterized as PATOS. In particular, emergency procedures, which are less amenable to quality improvement practices, were independently associated with PATOS SSI and were not associated with non-PATOS SSI. The results of this investigation support excluding PATOS SSIs from CMS reporting.

Our analysis identified several significant differences between procedures who developed PATOS versus non-PATOS COLO surgery SSIs. The COLO SSIs differed in surgical characteristics, including wound class, emergent procedure, endoscopic procedure, and surgical center volume. These differences are likely because emergent procedures were more commonly performed at high-volume centers (73.6%) and were open (74.5%) rather than laparoscopic procedures. Similarly, wounds that were categorized as contaminated or dirty were also more commonly seen at high-volume centers (81.5%). The EMM between contaminated or dirty wound class and emergent procedure was significant. These associations imply that patients who undergo colon procedures at high-volume hospitals are more likely to have features that are difficult to modify.

The definition of PATOS requires that evidence of infection be noted intraoperatively at the depth of subsequent SSI and documented within the narrative portion of the operative note. We found that only 102 (58%) of the PATOS COLO SSIs were associated with contaminated or dirty wounds. We expected that the categories of PATOS and contaminated or dirty wound class would have a larger amount of overlap. This finding raises questions about the accuracy of wound class data and/or application of the PATOS definition. At the very least, these data confirm that the PATOS definition continues to require review for specific documentation instead of inferring its presence based on wound class. To the best of our knowledge, this overlap has not been thoroughly evaluated in prior studies and may be the focus of a future prospective audit of wound class documentation.

Moreover, the non-PATOS COLO surgery SSIs and PATOS COLO surgery SSIs had different frequencies of polymicrobial and *Candida* spp infections. The reason for the difference in polymicrobial and *Candida* spp cultures between the 2 groups is unclear, but empiric antibiotic treatment for the patients with a suspected infection may influence the pathogens recovered from the surgical site. In other words, empiric broad-spectrum antibacterial agents for patients with PATOS COLO SSIs may explain why less bacterial gastrointestinal flora and more *Candida* spp are present in the PATOS group. Ultimately, additional studies are needed to answer this question.

Our study had several limitations. Our study is inherent to misclassification and selection bias given the retrospective nature of the analysis. In addition, some patients with complex COLO SSI may not have been identified if they presented to another hospital for infection treatment or if they presented outside the 30-day surveillance window. However, we limited our analysis to only complex SSIs and used the same methods and definitions for surveillance across 34 hospitals throughout the study period. Although most healthcare in the United States is provided in community hospitals, the generalizability of our findings to other settings may be limited. Also, we were unable to determine the differences in long-term outcomes of PATOS versus non-PATOS SSIs based on limitations of the database, but future studies may focus on these outcomes. Lastly, we were unable to include all SSI risk factors in our multivariate logistic regression model, such as prophylactic antibiotic, skin preparation agent, blood transfusion, etc, due to the limitations of the pre-established SSI database.

To date, few studies have described the characteristics of PATOS COLO surgery SSIs. Our analysis shows that PATOS COLO surgery SSIs have distinct characteristics from non-PATOS COLO surgery SSIs. Removal of PATOS COLO surgery SSIs from CMS reporting levels the playing field for hospitals that more frequently perform open and emergent procedures. Future studies are needed to identify more systematic strategies for identifying PATOS procedures and could address the preventability of PATOS COLO SSIs versus non-PATOS COLO SSIs.

## Figures and Tables

**Table 1.** Univariate Descriptive Statistics of All Colon Surgeries and All Complex Surgical Site Infections (SSIs)

Variable	All Colon Surgeries (N=28,188)	Complex SSIs (N=720)	Non-complex SSIs (N=27,468)	*P* Value Comparing SSIs and Non-SSIs
Age, median y, (SD)	62 (14)	59 (15)	64 (14)	<.01^a^
Age >75 y, no. (%)	5,106 (18.1)	90 (12.5)	5,061 (18.3)	<**.01**^b^
Sex, male, no (%)	13,157 (46.7)	356 (49.4)	12,801 (46.6)	.14^b^
BMI (kg/m2, median, Q1–Q3)	27.8 (23.9–32.4)	28.2 (24.1–32.9)	27.8 (23.9–32.4)	.63^a^
Obesity (BMI > 30, %)	9,306 (33.0)	257 (35.7)	9,049 (32.9)	.13^b^
Operation time, min (Q1–Q3)	131 (92–187)	157 (113–218)	130 (90–186)	.07^a^
Operation time >75th percentile = 187 min (%)	6,935 (24.6)	257 (35.7)	463 (2.2)	**.01** ^b^
Diabetes (%)	1,797 (7.3)	45 (7.2)	1,752 (7.3)	.81^b^
ASA 1–2 (low, %)	9,621 (34.1)	223 (31.0)	9,398 (34.2)	
ASA 3–5 (high, %)	18,070 (65.9)	497 (69.0)	18,070 (65.8)	.07^b^
Wound class (C, CC, %)	22,252 (78.9)	520 (72.2)	21,732 (79.1)	
Wound class (Co, D, %)	5,936 (21.1)	200 (27.8)	5,736 (20.9)	<**.01**^b^
Emergency (%)	2,774 (9.8)	120 (16.7)	2,654 (9.7)	<**.01**^b^
Open procedure (%)	15,951 (56.6)	450 (62.5)	15,501 (56.4)	**.01** ^b^
Hospital volume >500 procedures	24,237 (86.0)	635 (88.2)	23,602 (85.9)	.09^b^

Note. BMI, body mass index; ASA, American Society of Anesthesiologists. Bold indicates statistical significance.

a Unpaired *t* test.

b χ^2^ test.

**Table 2.** Univariate Descriptive Statistics Comparing Complex PATOS COLO SSis and Complex Non-PATOS COLO SSIs

Variable	Non-PATOS SSis (N=544)	PATOS SSIs (N=176)	*P* Value Comparing PATOS SSis and Non-PATOS SSis
Age, median y (SD)	59 (16)	60 (15)	.46
Age >75 y, no. (%)	74 (13.6)	16 (9.1)	.15
Male	273 (50.2)	83 (47.2)	.49^b^
BMI, median kg/m^2^ (Q1–Q3)	28.3 (24.1–33.1)	28.0 (24.2–32.6)	.72^a^
Obesity (BMI > 30), no. (%)	191 (35.1)	66 (37.5)	.59^b^
Operation time, min (Q1–Q3)	160 (116–222)	148 (104.5–202.5)	.08^a^
Operation time >75th percentile = 187 min (%)	203 (37.3)	54 (30.7)	.12^b^
Diabetes mellitus (%)	36 (8.0)	9 (5.1)	.23^b^
ASA 1–2 (low, %)	176 (32.4)	47 (26.7)	
ASA 3–5 (high, %)	368 (67.6)	129 (73.3)	.19^b^
Wound class (C, CC, %)	446 (82.0)	74 (42.1)	
Wound class (Co, D, %)	98 (18.0)	102 (58.0)	<**.01**^b^
Emergency (%)	59 (10.9)	61 (34.7)	<**.01**^b^
Open procedure (%)	328 (58.5)	132 (75.0)	<**.01**^b^
Hospital volume >500 procedures	471 (86.6)	164 (93.2)	**.02** ^b^

Note. PATOS, present at the time of surgery; COLO, colon surgical procedure; SSI, surgical site infection; BMI, body mass index; ASA, American Society of Anesthesiologists. Bold indicates statistical significance.

a Unpaired *t* test.

b χ^2^ test.

**Table 3.** Variable Analysis for Complex COLO SSis Using Multivariable Logistic Regression Models^a^

Variable	Model 1 Odds Ratio (N=720)	*P* Value	Model 2 Odds Ratio (N=544)	*P* Value	Model 3 Odds Ratio (N=176)	*P* Value
Age >75 y (%)	**1.56 (1.23–1.96)**	<**.01**	**1.37 (1.06–1.75)**	**.01**	1.39 (0.93–2.13)	.10
Sex, male	1.08 (0.51–1.25)	.29	1.13 (0.96–1.33)	.15	1.00 (0.82–1.24)	.96
Obesity, BMI > 30 (%)	1.04 (0.89–1.21)	.61	1.02 (0.86–1.22)	.80	1.04 (0.85–1.28)	.69
Operation time >75th percentile = 187 min (%)	**1.75 (1.50–2.04)**	<**.01**	**1.82 (1.53–2.16)**	<**.01**	1.13 (0.85–1.52)	.40
Diabetes mellitus diagnosis (%)	0.93 (0.68–1.25)	.61	1.00 (0.71–1.40)	.99	0.85 (0.48–1.52)	.59
ASA score 3–5 (%)	1.17 (1.00–1.38)	.05	1.15 (0.96–1.39)	.13	0.94 (0.73–1.39)	.61
Dirty or contaminated wound class (%)	**1.27 (1.07–1.50)**	**.01**	**1.28 (1.03–1.60)**	**.03**	**2.80 (2.12–3.71)**	<**.01**
Emergency surgery (%)	**1.87 (1.53–2.29)**	<**.01**	1.28 (0.97–1.68)	.08	**1.88 (1.45–2.44)**	<**.01**
Open procedures (%)	**1.20 (1.03–1.39)**	**.02**	1.11 (0.93–1.32)	.23	1.30 (0.98–1.72)	.07
Hospital volume >500 procedures during study period	**1.35 (1.08–1.69)**	**.01**	1.05 (0.82–1.35)	.67	**2.02 (1.22–3.33)**	**.01**

Note. COLO, colon surgical procedure; SSI, surgical site infection; BMI, body mass index; ASA, American Society of Anesthesiologists; PATOS, present at the time of surgery. Bold indicates statistical significance.

a The outcome of model 1 was complex COLO SSis. The outcome of model 2 was non-PATOS complex COLO SSis. The outcome of model 3 was PATOS complex COLO SSis.

**Table 4.** Comparison of Pathogens That Caused Complex Non-PATOS SSis and Complex PATOS SSIs

Organism	Isolates From Complex SSis, No. (%)	Isolates From Non-PATOS SSIs, No. (%)^a^	Isolates From PATOS SSIs No. (%)^b^
*Escherichia coli*	257 (36)	187 (34)	60 (34)
*Enterococcus* spp	177 (25)	135 (25)	42 (24)
No pathogen identified	138 (19)	105 (19)	33 (19)
***Klebsiella* spp**	**63 (9)**	**57 (10)**	**6 (3)**
***Candida* spp**	**64 (9)**	**38 (7)**	**26 (15)**
***Bacteroides* spp**	**51 (7)**	**47 (9)**	**4 (2)**
*Streptococcus* spp	37 (5)	28 (5)	9 (5)
*Staphylococcus aureus*	33 (5)	27 (5)	6 (3)
*Pseudomonas* spp	28 (4)	21 (4)	7 (5)
*Proteus* spp	20 (3)	18 (3)	2 (1)
*Enterobacter* spp	20 (3)	16 (3)	4 (2)
*Clostridium* spp	19 (3)	11 (2)	8 (5)
*Citrobacter* spp	17 (2)	16 (3)	1 (1)
Coagulase-negative staphylococci	14 (2)	9 (2)	5 (3)
**Polymicrobial infection**	**437 (61)**	**378 (69)**	**59 (33)**

Note. PATOS, present at the time of surgery; SSI, surgical site infection.

a % based on 544 non-PATOS SSis.

b % based on 176 PATOS SSis.
